# Cyclin D1 Serves as a Poor Prognostic Biomarker in Stage I Gastric Cancer

**DOI:** 10.3390/cimb44030093

**Published:** 2022-03-20

**Authors:** Se-Il Go, Gyung Hyuck Ko, Won Sup Lee, Jeong-Hee Lee, Sang-Ho Jeong, Young-Joon Lee, Soon Chan Hong, Woo Song Ha

**Affiliations:** 1 Department of Internal Medicine, Gyeongsang National University Changwon Hospital, Institute of Health Sciences, Gyeongsang National University College of Medicine, Changwon 51472, Korea; gose1@gnuh.co.kr; 2 Department of Pathology, Institute of Health Sciences, Gyeongsang National University College of Medicine, Jinju 52727, Korea; gyunghko@gnu.ac.kr (G.H.K.); jhlee7@gnu.ac.kr (J.-H.L.); 3 Department of Internal Medicine, Institute of Health Sciences, Gyeongsang National University College of Medicine, Jinju 52727, Korea; 4 Department of Surgery, Gyeongsang National University Changwon Hospital, Institute of Health Sciences, Gyeongsang National University College of Medicine, Changwon 51472, Korea; shjeong@gnu.ac.kr (S.-H.J.); yjlee@gnu.ac.kr (Y.-J.L.); 5 Department of Surgery, Institute of Health Sciences, Gyeongsang National University College of Medicine, Jinju 52727, Korea; hongsc@gnu.ac.kr (S.C.H.); woosongha@gnu.ac.kr (W.S.H.)

**Keywords:** cyclin D1, epidermal growth factor receptor (EGFR), stage I gastric cancer, early gastric cancer, node-negative gastric cancer

## Abstract

TNM stage still serves as the best prognostic marker in gastric cancer (GC). The next step is to find prognostic biomarkers that detect subgroups with different prognoses in the same TNM stage. In this study, the expression levels of epidermal growth factor receptor (EGFR) and cyclin D1 were assessed in 96 tissue samples, including non-tumorous tissue, adenoma, and carcinoma. Then, the prognostic impact of EGFR and cyclin D1 was retrospectively investigated in 316 patients who underwent R0 resection for GC. EGFR positivity increased as gastric tissue became malignant, and cyclin D1 positivity was increased in all the tumorous tissues. However, there was no survival difference caused by the EGFR positivity, while the cyclin D1-postive group had worse overall survival (OS) than the cyclin D1-negative group in stage I GC (10-year survival rate (10-YSR): 62.8% vs. 86.5%, *p* = 0.010). In subgroup analyses for the propensity score-matched (PSM) cohort, there were also significant differences in the OS according to the cyclin D1 positivity in stage I GC but not in stage II and III GC. Upon multivariate analysis, cyclin D1 positivity was an independent prognostic factor in stage I GC. In conclusion, cyclin D1 may be a useful biomarker for predicting prognosis in stage I GC.

## 1. Introduction

Gastric adenocarcinoma, commonly referred to as gastric cancer (GC), is the third most common cause of cancer death worldwide [1] and the second most common cause of cancer death in Korea [2]. Tremendous efforts have been made to detect predictive biomarkers [3], but the pathologic TNM stage still serves as one of the best prognostic markers in GC. The TNM stages are determined from I to IV according to the depth of tumor invasion, regional lymph node metastasis, and distant metastasis. However, we know that heterogeneous subgroups with different prognoses exist within the same TNM stage; widespread metastasis is sometimes observed several months after the surgical resection of early gastric cancer (EGC) for which adjuvant chemotherapy is not applied, according to clinical guidelines. In addition, we previously demonstrated that heterogeneous subgroups with different prognoses exist in EGC by reporting that CD44 variant 9 and Ki-67 expression served as prognostic biomarkers in EGC [4,5]. Therefore, the next step is to find prognostic biomarkers that detect subgroups with different clinical features and prognoses within the same TNM stage. With a literature review, we also found that the poor prognostic factors in GC would exhibit rapid growth, metastasis, and drug resistance for GC [6,7,8]. We believe that the biomarkers that are associated with the above features are relevant to poor prognosis in GC.

Epidermal growth factor receptor (EGFR) is a member of the ErbB family of receptors, a subfamily of four closely related receptor tyrosine kinases. It is found to be overexpressed in various cancers, including colorectal cancer, pancreatic cancer, and GC [9,10,11,12]. High EGFR expression is associated with an increased risk of metastasis and drug resistance, and the inhibition of EGFR leads to a reduction in cancer migration and angiogenesis and an increase in drug sensitivity in cancers [13,14]. In addition, EGFR gene amplification is related to lymph node metastases in GC [15]. Therefore, we thought that EGFR expression deserves to be investigated to determine whether it is a biomarker in GC.

The next candidate is cyclin D1, a protooncogene that plays a positive regulation role in cancer progression [16]. Increased cyclin D1 expression is an early cell proliferation event that is stimulated by growth factors or other mitogens [17]. Cyclin D1 has previously been used as a biomarker for cell proliferation and prognosis in various types of cancer as much as the Ki-67 proliferation index has [18,19,20,21]. We considered it worthwhile to investigate the role of cyclin D1 as a prognostic biomarker in GC.

Therefore, we planned this study to determine whether EGFR and the cyclin D1 protein serve as prognostic biomarkers in GC. In addition, subgroup analysis and propensity score-matched analysis were conducted to identify a specific group in which the biomarkers could be applied usefully.

## 2. Materials and Methods

### 2.1. Patients and Specimens

First, as a pilot study to evaluate the associations between the biomarker expression and tumor development, 96 tissue samples including 23 non-tumorous tissues (6 normal mucosa, 5 *Helicobacter pylori*-related gastritis, and 12 intestinal metaplasia), 24 adenoma (12 low-grade adenoma and 12 high-grade adenoma), and 49 GC (23 EGC and 26 advanced gastric cancer (AGC)) were used. Then, we retrospectively reviewed 316 consecutive patients who underwent R0 resection for GC from 2004 to 2013 at a single institution. Patients who underwent R1/R2 resection or endoscopic mucosal resection and had metastasis to a distant organ were excluded. The demographics, clinical data, and histologic findings were obtained through an electronic medical record review. The TNM stage was classified according to the 8th edition AJCC staging system for gastric cancer. Histologic classification was performed using the WHO classification (G1 well differentiated, G2 moderately differentiated, and G3 poorly differentiated and undifferentiated) and Lauren classification (intestinal, diffuse, and mixed). EGC was defined as a tumor confined to the mucosa or submucosa regardless of the presence of lymph node metastases. AGC was defined as a tumor invading the muscularis propria or deeper layers.

The expression levels of each biomarker were determined by immunohistochemical (IHC) staining of tissue microarray (TMA) sections. A 2 mm diameter core tissue in each case was arrayed in a new recipient paraffin block. The TMA section of each slide was then deparaffinized, rehydrated, and incubated in 3% H_2_O_2_ to prevent non-specific background staining. After heating in a microwave oven at 700 W for 20 min with 10 mmol/L citrate buffer (pH 6.0) and incubating for 10 min with Ultra V Block (Lab Vision, Fremont, CA, USA) at room temperature, slides were incubated for 32 and 44 min with primary monoclonal antibodies specific to Cyclin D1 and EGFR, respectively. The BenchMark XT (VENTANA, Tucson, AZ, USA) was used for IHC staining. The expression level of the biomarkers was scored and interpreted by two pathologists blind to the patients’ clinical data as follows: negative (<1% of tumor cells were stained) and positive (≥1% of tumor cells were stained; 1+ (1–20%), 2+ (21–50%), and 3+ (>50%)) (Figure S1, see Supplementary Materials).

### 2.2. Statistical Analysis

Overall survival (OS) was calculated as the time from surgery to cancer-related death or last follow-up. Missing data on the date and cause of death in electronic medical records were obtained from the National Statistical Office of Korea [21]. A Kaplan–Meier curve was plotted for survival. A log-rank test was performed to compare the survival probability. The median follow-up duration was calculated by the reverse Kaplan–Meier method. The Cox regression model was used to analyze multiple variables influencing patient survival. The final model was internally validated by bootstrap resampling (200 replications). If there was a discrepancy in the variables listed as the baseline characteristics of patients between the positive and negative groups for each biomarker, propensity score matching (PSM) was performed to reduce the probability of selection bias. The expression of the biomarkers (negative vs. positive) was regressed by a logistic regression analysis for the conventional prognostic factors as follows: depth of invasion, nodal status, TNM stage, histologic differentiation, and Lauren classification. A nearest-neighbor matching algorithm with a 1:1 ratio was applied to the PSM. A two-sided *p*-value < 0.05 was considered significant. The MatchIt package in R software version 4.0.5 (The R Foundation for Statistical Computing, Vienna, Austria) was used for the PSM. All other statistical analyses were performed with the Stata software version 16.1 (Stata Corp., College Station, TX, USA).

## 3. Results

### 3.1. Baseline Characteristics

The baseline characteristics in the unmatched cohort are presented in Table 1. In an unmatched cohort of 316 patients, the median age of patients was 65 years (interquartile range (IQR), 56–70). The male-to-female ratio was about 2 to 1. The most frequent type of surgery was subtotal gastrectomy (221/316, 69.9%), as the majority of the primary tumors were located in the lower third of the stomach (215/316, 68.0%). The number of patients with EGC and AGC was the same (158:158). Node-positive diseases were observed in 122 of 316 (38.6%) patients. The proportion of poorly differentiated histology in the WHO classification and intestinal type in the Lauren classification was higher than other histologic subtypes.

The cyclin D1 positivity was 19.3% (61 of 316). Fifty-seven and four patients had 1+ and 2+ cyclin D1 expressions, respectively. None of the patients had 3+ cyclin D1 expression. Patients were evenly distributed overall with respect to the variables presented in the baseline characteristics, except that the cyclin D1-negative group tended to have a more advanced stage (*p* = 0.088). EGFR positivity was 13.0% (41 of 316). Thirty-three, two, and six patients had 1+, 2+, and 3+ expressions of EGFR, respectively. There were no significant differences in the baseline characteristics between the EGFR-positive and EGFR-negative groups. These findings suggest that cyclin D1 and EGFR expression positivity were relatively low in GC and that there was an imbalance in terms of the cancer stage between the cyclin D1-positive and cyclin D1-negative groups.

### 3.2. The Expression Patterns of Each Biomarker According to the Progression of Carcinogenesis and Advancement of Malignancy in GC

**Figure 1:**
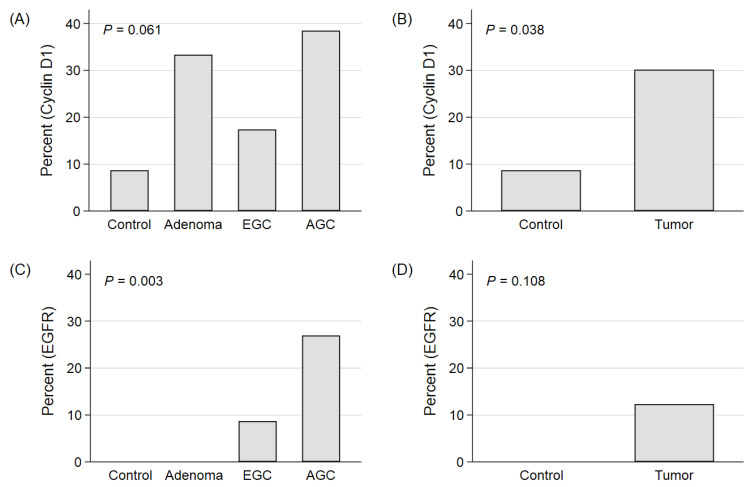
Expression of the biomarkers according to the progression of carcinogenesis. (**A**,**B**) Cyclin D1 and (**C**,**D**) EGFR.

The expression patterns of each biomarker are presented in Figure 1, which revealed that EGFR expression increased with the progression of carcinogenesis and the advancement of malignancy, whereas cyclin D1 expression was increased in all tumorous tissues (adenoma, EGC, and AGC) compared to normal tissues and did not increase with advancement from EGC to AGC. The positive expression rates of cyclin D1 were 8.7% (2/23), 33.3% (8/24), 17.4% (4/23), and 38.5% (10/26) in the control tissue, adenoma, EGC, and AGC, respectively (*p* = 0.061). In the tumor group (adenoma, EGC, and AGC), the positive expression rate of cyclin D1 was high compared to the control group (non-tumorous tissue) (30.1% (22/73) vs. 8.7% (2/23), *p* = 0.038). The positive expression rates of EGFR were 0%, 0%, 8.7% (2/23), and 26.9% (7/26) in the control tissue, adenoma, EGC, and AGC, respectively (*p* = 0.003). These findings indicate that EGFR expression positivity increased as the gastric tissue became malignant and that cyclin D1 expression positivity was increased in all of the tumorous tissues.

### 3.3. Overall Survival According to Cyclin D1 and EGFR Expression in Whole Cohort

**Figure 2:**
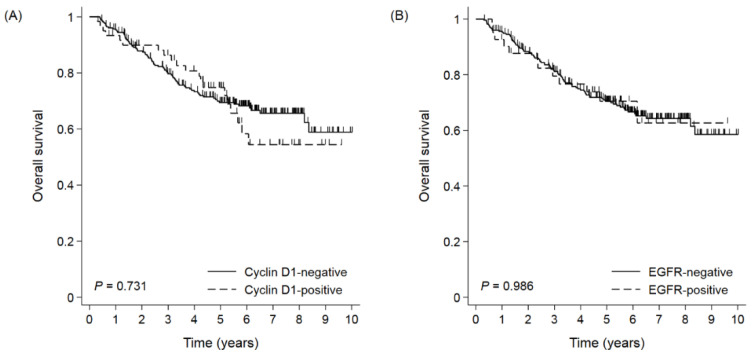
Overall survival by (**A**) cyclin D1 and (**B**) EGFR positivity in total cohort.

**Figure 3:**
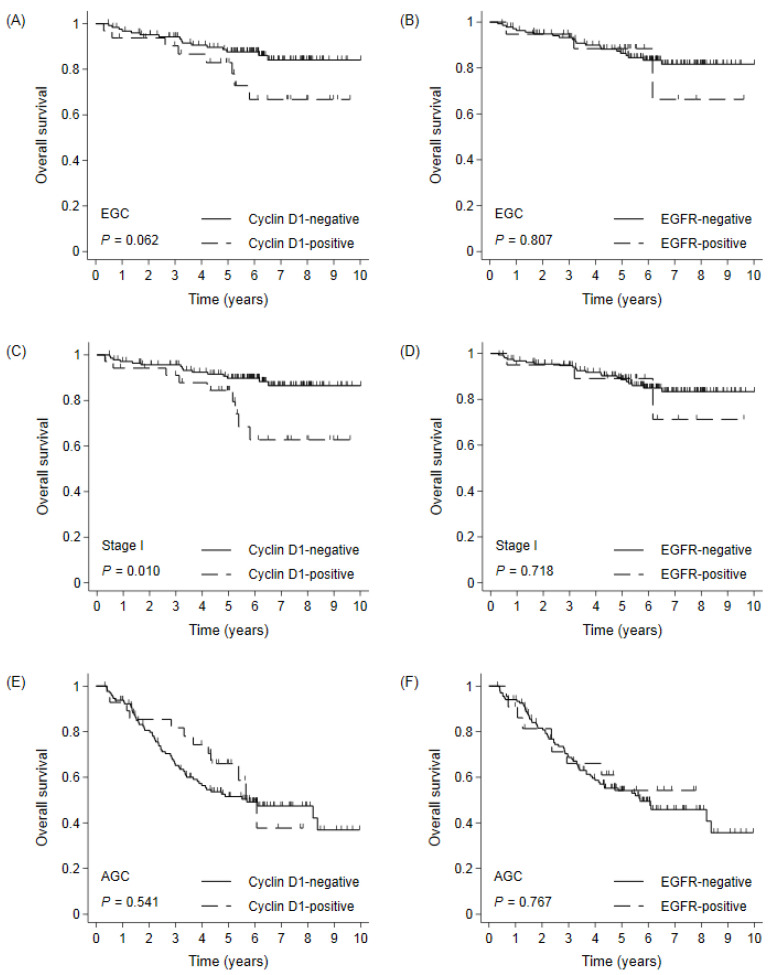
Overall survival by (**A**,**C**,**E**) cyclin D1 and (**B**,**D**,**F**) EGFR positivity in EGC, stage I gastric cancer, and AGC. EGC: early gastric cancer; AGC: advanced gastric cancer.

**Figure 4:**
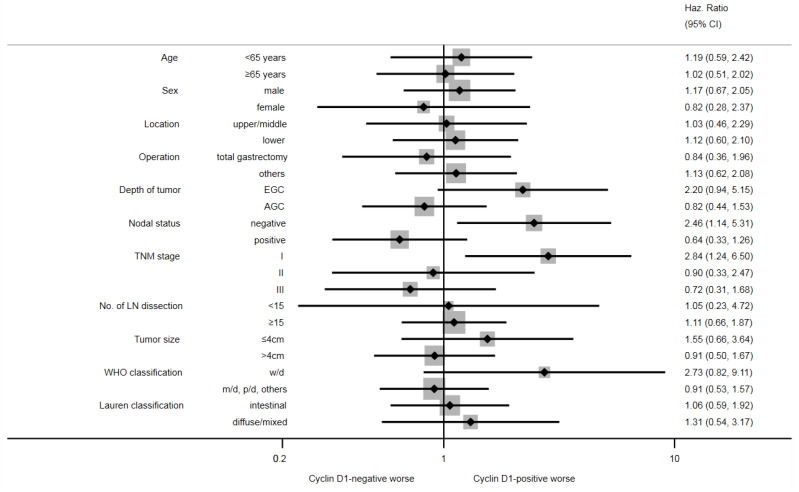
Forest plot for subgroup analyses of overall survival by cyclin D1 positivity.

The median follow-up duration was 73 months. In the whole cohort, the median OS was not reached, and the 5- and 10-YSRs were 70.4% and 58.7%, respectively. When survival was compared according to the expression of each biomarker, there were no differences in the OS between the negative and positive expression groups for each biomarker (Figure 2). In subgroup analyses, the cyclin D1-positive group had shorter survival than the cyclin D1-negative group in stage I GC (Figure 3). The 5- and 10-YSRs were 89.8% and 86.5% in the cyclin D1-negative group and 84.4% and 62.8% in the cyclin D1-positive group in stage I GC, respectively (*p* = 0.010, Figure 3C). In EGC, the cyclin D1-negative group had better survival than cyclin D1-positive with borderline statistical significance (*p* = 0.062, Figure 3A), but not in AGC (*p* = 0.541, Figure 3E). However, the EGFR positivity did not affect the survival in any subgroup (Figure 3B,D,F). On a forest plot, the cyclin D1-positive group showed a poor prognosis compared to the cyclin D1-negative group in stage I and in node-negative disease (Figure 4). Upon multivariate analysis in stage I GC, cyclin D1 positivity was an independent poor prognostic factor (hazard ratio (HR) 2.801, 95% confidence interval (CI) 1.221–6.426, *p* = 0.015, Table 2)).

### 3.4. Survival According to Cyclin D1 Expression in Propensity Score-Matched Cohort

**Figure 5:**
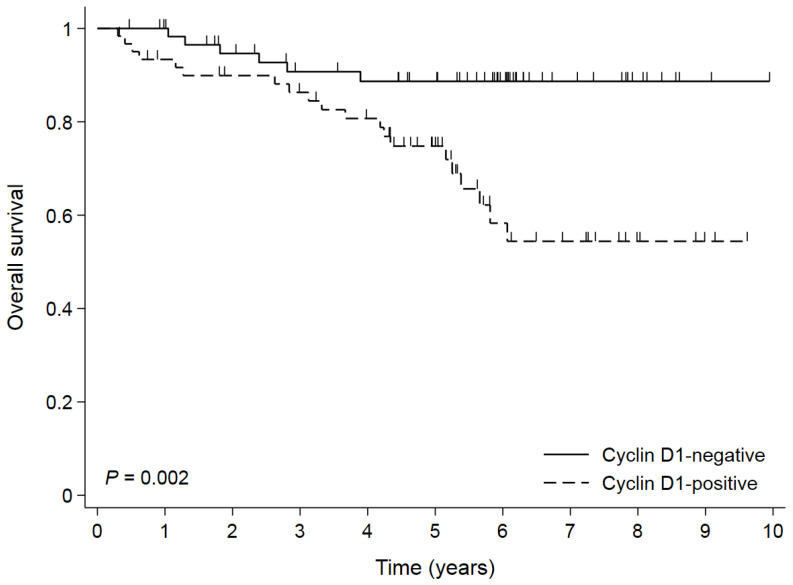
Overall survival by cyclin D1 positivity in propensity score-matched cohort.

**Figure 6:**
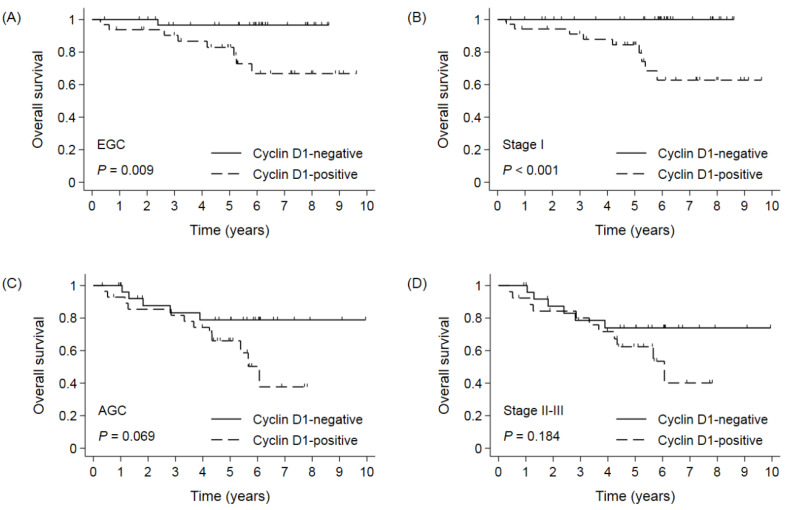
Overall survival by cyclin D1 in (**A**) EGC, (**B**) stage I gastric cancer, (**C**) AGC, and (**D**) stage II-III gastric cancer in propensity score-matched cohort. EGC: early gastric cancer; AGC: advanced gastric cancer.

**Figure 7:**
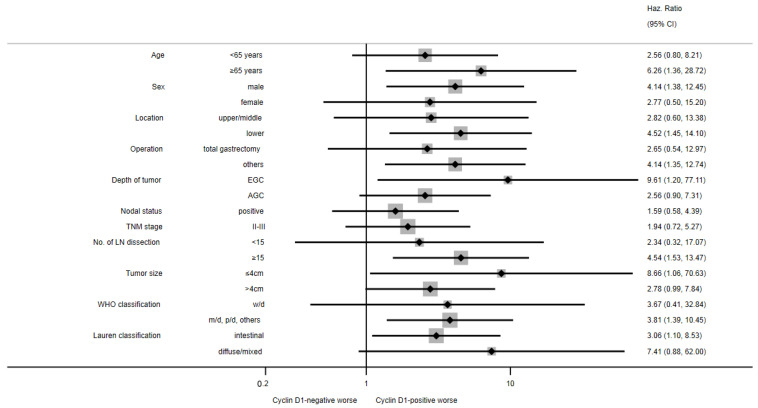
Forest plot for subgroup analyses of overall survival by cyclin D1 positivity in propensity score-matched cohort.

Given the imbalance in tumor stages among the basic characteristics of patients the between cyclin D1-positive and -negative groups, survival was reassessed between these two groups in the PSM cohort. After PSM, tumor stage and histologic classification were well balanced between the cyclin D1-negative and -positive groups (Table S1 and Figure S2). In 122 patients from the PSM cohort, the 5- and 10-YSRs were 88.7% and 88.7% in the cyclin D1-negative group and 74.8% and 54.4% in the cyclin D1-positive group, respectively (*p* = 0.002, Figure 5). In subgroup analyses, similarly to the result in the pre-PSM cohort (whole cohort), the cyclin D1-positive group had a poor prognosis compared to the cyclin D1-negative group in patients with EGC or stage I GC (Figure 6A,B), while there were no differences in the OS in patients with AGC or stage II-III GC (Figure 6C,D). Overall, the cyclin D1-positive group had a worse prognosis than the cyclin D1-negative group in the majority of subgroups (Figure 7). In a multivariate analysis of the PSM cohort, cyclin D1 expression was an independent prognostic factor (HR 3.630, 95% CI 1.450–9.086, *p* = 0.006). On bootstrap resampling, the statistical significance of the cyclin D1-positive group was internally validated (Table 3).

## 4. Discussion

This study was designed to find biomarkers that detect subgroups with different prognoses within the same stage. To accomplish this aim, we assessed the role of EGFR and cyclin D1 according to the depth of invasion and cancer stage. We also assessed each biomarker expression profile in normal cells, adenoma, EGC, and AGC to estimate the role of the biomarkers in gastric carcinogenesis. From the above results, we found that EGFR positivity was only observed in AGC (Figure 1). However, high EGFR expression failed to select a group with poor prognosis in AGC, while many previous studies reported that the overexpression of EGFR was related to tumor growth and drug resistance [13,22,23]. In addition, our result was different from previous data that suggested that EGFR amplification was associated with lymph node metastases [24,25,26]. This discrepancy can be explained. First, there are many signals that induce EGFR positivity in IHC, not just EGFR amplification [27,28,29]. Second, if the candidate biomarker is tested in the cohort including a subgroup in which the biomarker has no impact on predicting prognosis, the biomarker may come out as ineffective by reducing the statistical effect size [30]. This means that although EGFR positivity may play a role as a prognostic biomarker in a small subgroup, its role as a biomarker with statistical significance could be hidden due to the heterogeneity of the study group. Therefore, we also assessed the prognostic role of each biomarker’s role in various subgroups stratified by clinically important variables such as age, sex, location, operation type, depth of tumor, nodal status, number of lymph nodes dissected, tumor size, and histologic classification. However, EGFR did not serve as a prognostic biomarker in any subgroup even though EGFR prognostic roles were tested in various subgroups.

On the other hand, cyclin D1 positivity showed a role as a prognostic factor in the node-negative GC as well as in TNM stage I GC, while cyclin D1 positivity was increased in all of the tumorous tissues without any significant increase in the positivity with the progression of carcinogenesis (adenoma to AGC). The statistical significance of this result increased when the PSM was performed despite the decrease in the number of patients, which means a significant reduction in statistical power. Cyclin D1 expression is tightly regulated in normal cells but is overexpressed in various ways in cancer. The overexpression of cyclin D1 contributes to uncontrolled cell proliferation and plays a central role in cancer carcinogenesis [16,17]. The overexpression of cyclin D1 has already been used as a cell proliferation and prognosis-related biomarker in several tumors together with the Ki-67 proliferation index [31,32]. The Ki-67 proliferation index also serves as a prognostic biomarker in EGC [4,33]. In addition, increased expression of Ki-67 and cyclin D1 has been reported to be associated with the development of precancerous lesions such as pancreatic intraepithelial neoplasia [34]. Therefore, in stage I GC, the proliferation index is considered to play an important role in the prognosis.

The strength of the study is that we assessed the EGFR and cyclin D1 expression profile from normal cells to cancer cells and then investigated its prognostic capability in GC within the same TNM stage. In previous studies suggesting the association of cyclin D1 expression with prognosis in GC, the prognostic impact of cyclin D1 by the TNM stage was not reported [18,35]. In addition, to find the hidden predictive role of the biomarkers, we also assessed the prognostic role of each biomarker in various subgroups. In contrast, there are several limitations in the study. First, this study is retrospectively designed. To overcome the potential bias caused by the retrospective study design, we additionally analyzed the PSM cohort and internally validated the multivariate Cox regression model using bootstrap resampling. Second, our criteria for the positive expression of each biomarker have not been validated in other studies. There are no standard criteria, and various criteria indicating positive expression of EGFR and cyclin D1 have been suggested [27,28,36,37]. A prospectively designed study using various criteria for positive expression of the biomarkers is warranted to confirm our results.

In conclusion, this study suggests that cyclin D1, but not EGFR, can be a useful biomarker in predicting the prognosis of stage I GC. Our results raise the question of whether adjuvant therapy is needed in those with positive cyclin D1.

## Figures and Tables

**Table 1:** Baseline characteristics of patients.

	Cyclin D1-Negative (*n* = 255)	Cyclin D1-Positive (*n* = 61)	*p*	EGFR-Negative (*n* = 275)	EGFR-Positive (*n* = 41)	*p*
**Median (IQR) age, years**	65 (56–70)	64 (54–70)	0.890	65 (56–70)	66 (56–69)	0.958
**Sex**			0.504			0.544
**Male**	164 (64.3)	42 (68.9)		181 (65.8)	25 (61.0)	
**Female**	91 (35.7)	19 (31.2)		94 (34.2)	16 (39.0)	
**Location**			0.117			0.997
**Upper**	26 (10.2)	12 (19.7)		33 (12.0)	5 (12.2)	
**Middle**	51 (20.0)	12 (19.7)		55 (20.0)	8 (19.5)	
**Lower**	178 (69.8)	37 (60.7)		187 (68.0)	28 (68.3)	
**Operation**			0.957			0.699
**Subtotal gastrectomy**	180 (70.6)	41 (67.2)		190 (69.1)	31 (75.6)	
**Total gastrectomy**	60 (23.5)	16 (26.2)		67 (24.4)	9 (22.0)	
**Proximal gastrectomy**	12 (4.7)	3 (4.9)		14 (5.1)	1 (2.4)	
**Wedge resection**	3 (1.2)	1 (1.6)		4 (1.5)	0	
**Depth of invasion**			0.669			0.616
**EGC**	126 (49.4)	32 (52.5)		139 (50.6)	19 (46.3)	
**AGC**	129 (50.6)	29 (47.5)		136 (49.5)	22 (53.7)	
**Nodal status**			0.650			0.455
**N0**	155 (60.8)	39 (63.9)		171 (62.2)	23 (56.1)	
**N+**	100 (39.2)	22 (36.1)		104 (37.8)	18 (43.9)	
**AJCC 8th edition staging**			0.088			0.577
**I**	141 (55.3)	35 (57.4)		156 (56.7)	20 (48.8)	
**II**	43 (16.9)	16 (26.2)		51 (18.6)	8 (19.5)	
**III**	71 (27.8)	10 (16.4)		68 (24.7)	13 (31.7)	
**Tumor size**			0.819			0.367
**≤** **4 cm**	117 (45.9)	27 (44.3)		128 (46.6)	16 (39.0)	
**>4 cm**	138 (54.1)	34 (55.7)		147 (53.5)	25 (61.0)	
**WHO classification**			0.597			0.389
**Well-differentiated**	56 (22.0)	10 (16.4)		60 (21.8)	6 (14.6)	
**Moderately differentiated**	77 (30.2)	21 (34.4)		82 (29.8)	16 (39.0)	
Poorly differentiated and others *	122 (47.8)	30 (49.2)		133 (48.4)	19 (46.3)	
**Lauren classification**			0.287			0.096
**Intestinal**	179 (70.2)	47 (77.1)		192 (69.8)	34 (82.9)	
**Diffuse or mixed**	76 (29.8)	14 (23.0)		83 (30.2)	7 (17.1)	

IQR: interquartile range; EGC: early gastric cancer; AGC: advanced gastric cancer; AJCC: American Joint Committee on Cancer. * Undifferentiated and mucinous adenocarcinoma as well as signet-ring cell carcinoma were included.

**Table 2:** Cox regression for overall survival in stage I gastric cancer.

	Univariate			Multivariate			
	HR	95% CI	*p*	HR	95% CI	*p*	*p* (Bootstrap)
Age (≥65 vs. <65)	2.623	1.087–6.328	0.032	2.679	1.110–6.466	0.028	0.042
Sex (male vs. female)	3.384	1.009–11.347	0.048	3.547	1.056–11.909	0.041	0.876
Location (upper/middle vs. lower)	1.086	0.465–2.538	0.849				
Operation (total gastrectomy vs. others)	1.894	0.752–4.774	0.176				
Depth of invasion (AGC vs. EGC)	0.651	0.153–2.770	0.561				
Nodal status (positive vs. negative)	2.035	0.477–8.685	0.337				
Tumor size (>4 cm vs. ≤4 cm)	0.687	0.273–1.730	0.425				
WHO classification (others vs. well-differentiated)	1.096	0.454–2.645	0.839				
Lauren classification (diffuse/mixed vs. intestinal)	1.055	0.419–2.658	0.910				
Cyclin D1 (positive vs. negative)	2.836	1.238–6.498	0.014	2.801	1.221–6.426	0.015	0.023
EGFR (positive vs. negative)	1.250	0.372–4.200	0.718				

HR: hazard ratio; CI: confidence interval; AGC: advanced gastric cancer; EGC: early gastric cancer.

**Table 3:** Cox regression for overall survival after PSM.

	Univariate			Multivariate			
	HR	95% CI	*p*	HR	95% CI	*p*	*p* (Bootstrap)
Age (≥65 vs. <65)	0.833	0.385–1.803	0.643				
Sex (male vs. female)	1.881	0.755–4.688	0.175				
Location (upper/middle vs. lower)	1.266	0.575–2.791	0.558				
Operation (total gastrectomy vs. others)	1.940	0.863–4.362	0.109				
Depth of invasion (AGC vs. EGC)	2.564	1.139–5.769	0.023	1.033	0.328–3.255	0.956	0.960
Nodal status (positive vs. negative)	3.847	1.736–8.526	0.001	3.169	1.014–9.906	0.047	0.077
Tumor size (>4 cm vs. ≤4 cm)	2.102	0.913–4.838	0.081	1.403	0.576–3.414	0.456	0.479
WHO classification (others vs. well-differentiated)	0.931	0.351–2.469	0.885				
Lauren classification (diffuse/mixed vs. intestinal)	1.313	0.551–3.126	0.539				
Cyclin D1 (positive vs. negative)	3.831	1.532–9.578	0.004	3.630	1.450–9.086	0.006	0.030

HR: hazard ratio; CI: confidence interval; AGC: advanced gastric cancer; EGC: early gastric cancer.

## Data Availability

The data presented in this study are available on request from the corresponding author. The data are not publicly available due to their containing information that could compromise the privacy of research participants.

## Supplementary Materials

The following supporting information can be downloaded at: https://www.mdpi.com/article/10.3390/cimb44030093/s1, Figure S1: Representative findings of immunohistochemical staining for the biomarkers in gastric cancer cells (×200); Figure S2: Jitter plot to assess the distribution of propensity scores; Table S1: Baseline characteristics of patients after propensity score matching.
