# Supplementary material for: Cyclin D1 Serves as a Poor Prognostic Biomarker in Stage I Gastric Cancer
Source: Curr Issues Mol Biol. 2022 Mar 20;44(3):1395–406. doi: 10.3390/cimb44030093 (PMC8947299; doi:10.3390/cimb44030093)
Supplement: Supplementary file 1 [file cimb-44-00093-s001.zip › cimb-1640259-supplementary.pdf]

Table S1. Baseline characteristics of patients after propensity score matching.

	Cyclin D1-negative (*n*=61)	Cyclin D1-positive (*n*=61)	*p*
Median (IQR) age, years	64 (54-68)	64 (54-70)	0.608
Sex			0.258
Male	36 (59.0)	42 (68.9)	
Female	25 (41.0)	19 (31.2)	
Location			0.045
Upper	3 (4.9)	12 (19.7)	
Middle	13 (21.3)	12 (19.7)	
Lower	45 (73.8)	37 (60.7)	
Operation			0.454
Subtotal gastrectomy	43 (70.5)	41 (67.2)	
Total gastrectomy	12 (19.7)	16 (26.2)	
Proximal gastrectomy	6 (9.8)	3 (4.9)	
Wedge resection	0 (0.0)	1 (1.6)	
Depth of invasion			0.856
EGC	33 (54.1)	32 (52.5)	
AGC	28 (45.9)	29 (47.5)	
Nodal status			>0.998
N0	39 (63.9)	39 (63.9)	
N+	22 (36.1)	22 (36.1)	
AJCC 8th edition staging			>0.998
I	35 (57.4)	35 (57.4)	
II	16 (26.2)	16 (26.2)	
III	10 (16.4)	10 (16.4)	
Tumor size			0.586
≤4 cm	30 (49.2)	27 (44.3)	
>4 cm	31 (50.8)	34 (55.7)	
WHO classification			>0.998
Well-differentiated	10 (16.4)	10 (16.4)	
Moderately-differentiated	21 (34.4)	21 (34.4)	
Poorly-differentiated and others*	30 (49.2)	30 (49.2)	

Lauren classification			>0.998
Intestinal	47 (77.1)	47 (77.1)	
Diffuse or mixed	14 (23.0)	14 (23.0)	

IQR: interquartile range; EGC: early gastric cancer; AGC: advanced gastric cancer; AJCC: American Joint Committee on Cancer.

*Undifferentiated and mucinous adenocarcinoma, and signet-ring cell carcinoma were included.

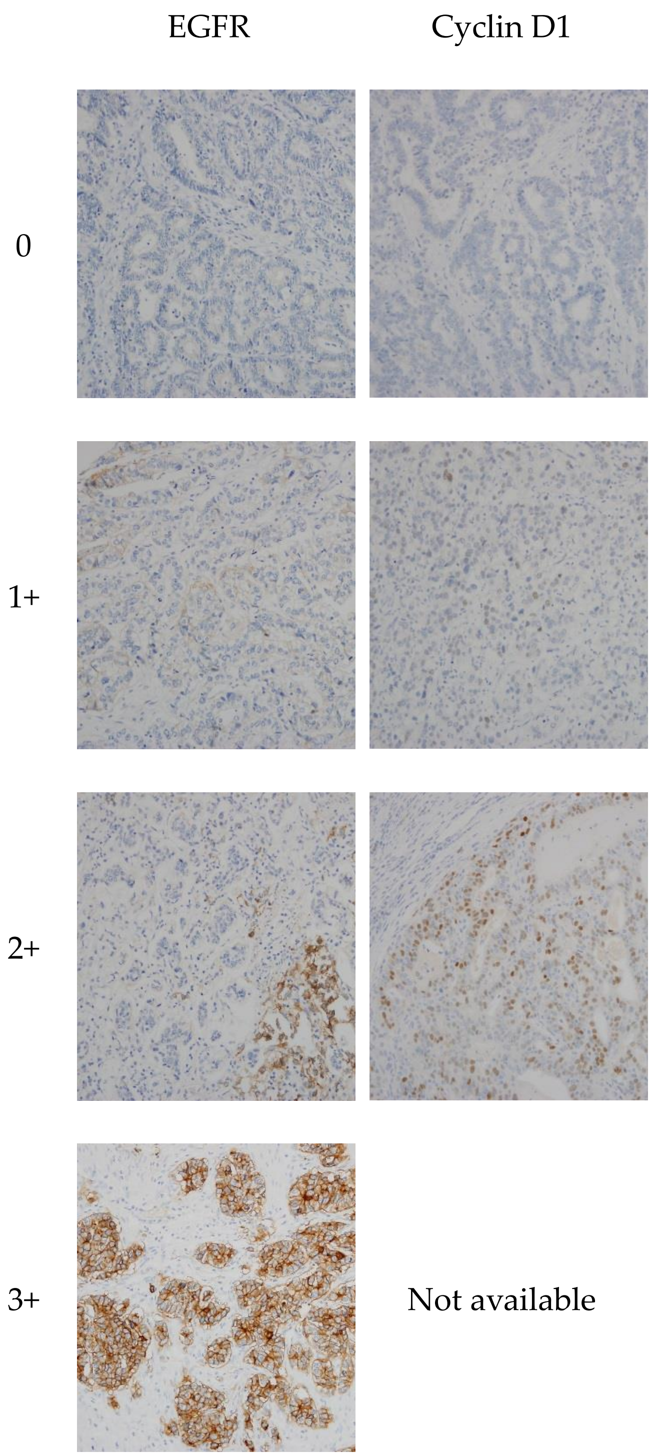

Figure S1. Representative findings of immunohistochemical staining for the biomarkers in gastric cancer cells (×200). The definitions of 0 (negative), 1+, 2+ and 3+ were <1%, 1-20%, 21-50%, and >50% of tumor cells stained, respectively.

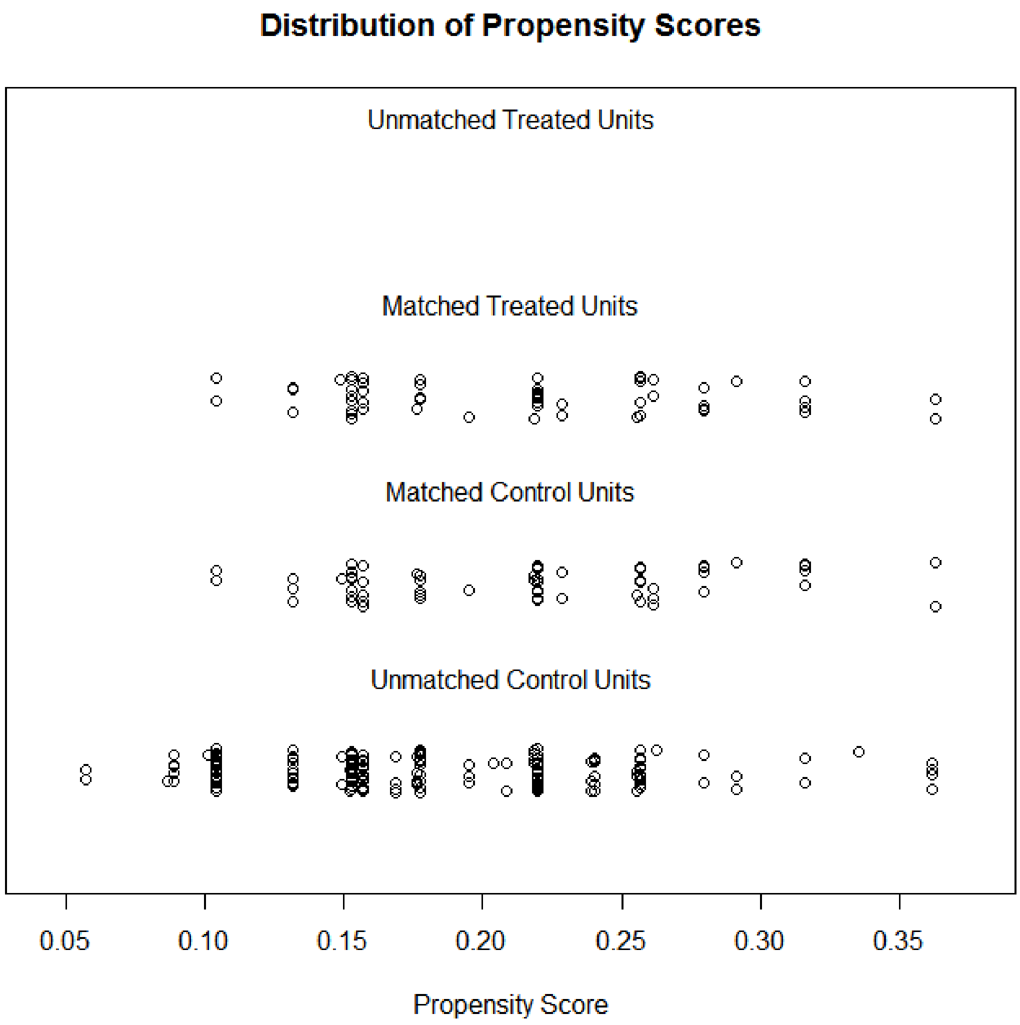

Figure S2. Jitter plot to assess the distribution of propensity scores.
